# A Text-Book of the Diseases of Women

**Published:** 1901-09

**Authors:** 


A Text-Book of the Diseases of Women. By Henry J. Garrigues, A. M., M. D., Gynecologist to St. Mark's Hospital, New York; to the German Dispensary, New York, etc. With 367 illustrations. Third edition. Thoroughly revised. Pp., 756; price, $4.50. Philadelphia: W. B. Saunders & Co. 1900.

This work has become a popular text-book throughout the country. A great many of the schools of any prominence have adopted it as a work on the diseases of women. Still, when compared with Thomas's work on the same subject, it falls far short in the arrangement and systematic discussion of the subject matter. Thomas, of course, is out of date, behind the times, but in the features mentioned, many of the present day authors could well afford to imitate. T. J. B.

